# Retrospective evaluation of a funnel-shaped mesh for the prevention of parastomal hernias in patients with permanent end colostomy

**DOI:** 10.1007/s10029-025-03481-x

**Published:** 2025-09-23

**Authors:** Francesco Brucchi, Maria Rennis, Pietro Achilli, Lorenzo Morini, Pietro Carnevali, Matteo Origi, Gianlorenzo Dionigi, Giovanni Ferrari

**Affiliations:** 1 General Surgery Residency Program, University of Milan, Milan, Italy; 2 Division of Surgery, Istituto Auxologico Italiano, Istituto di Ricovero e Cura a Carattere Scientifico (IRCCS), Milan, Italy; 3 Department of Mini-Invasive Surgery, ASST Grande Ospedale Metropolitano Niguarda, Milan, Italy; 4 Department of Pathophysiology and Transplantation, University of Milan, Milan, Italy

**Keywords:** Parastomal hernia prevention, Funnel-shaped mesh, Permanent end colostomy, Colorectal surgery, Prophylactic mesh placement, Long-term outcomes

## Abstract

**Background:**

Parastomal hernia (PSH) is the most common long-term complication after abdominoperineal resection (APR) with permanent end colostomy. Although prophylactic mesh placement has been suggested to reduce the rate of PSH, the optimal mesh type and surgical technique remain unclear. Recently, three-dimensional funnel-shaped meshes have been introduced to stabilize the bowel loop and minimize stoma-related mechanical stress, potentially reducing the incidence of PSH.

**Methods:**

This retrospective cohort study, reported in accordance with STROBE guidelines, included consecutive patients who underwent elective laparoscopic permanent end colostomy (PEC) between 2011 and 2019 at a single institution. Patients were assigned to either a group without mesh or a group that received prophylactic intraperitoneal funnel-shaped mesh. The primary endpoint was radiologically confirmed PSH incidence. Kaplan-Meier analysis and Cox regression were used to assess differences in cumulative PSH risk over time.

**Results:**

Seventy-five patients were included (mesh group: 37; no-mesh group: 38), with a median follow-up of 46 and 43 months, respectively. The mesh group had a lower, but not statistically significant, absolute incidence of PSH (21.6% vs. 39.5%, p = 0.094). Importantly, the Kaplan-Meier analysis revealed a significantly lower cumulative incidence of PSH in the mesh group over time (p= 0.033). Postoperative complication rates were comparable between the groups.

**Conusions:**

Prophylactic placement of a funnel-shaped mesh during PEC was associated with a reduced cumulative incidence of PSH over long-term followup without increasing surgical morbidity. These results underline the potential benefit of funnel-shaped meshes in PSH prevention and highlight the need for prospective randomized studies.

## Introduction

Pernent end colostomy (PEC) creation after abdominoperineal resection (APR) is associated with several complications, among which parastomal hernia (PSH) represents the most common late complication [ma^1^, ^2^]. According to the European Hernia Society guidelines, a PSH is defined as any abnormal protrusion of intra-abdominal contents through the abdominal wall defect created for stoma placement [3]. Reported incidence rates for PSH vary widely, ranging from 25 to 80%, largely due to differences in follow-up duration, patient characteristics and diagnostic criteria used in different studies [4].

Preventing PSH is essential to improving patient outcomes and reducing healthcare costs. PSH often requires additional surgical interventions, which are associated with significant risks and further strain healthcare resources [5]. In response, various prophylactic strategies have been explored, including technical refinements in stoma construction and the use of prophylactic mesh to reinforce the abdominal wall at the stoma site [6].

Various approaches to prophylactic mesh placement have been described over the past two decades, each with different advantages and limitations in terms of complication profile and patient selection. Despite the EHS recommendations favouring mesh insertion at initial stoma placement, particularly in high-risk patients, there is still no consensus on the optimal technique or mesh type, and clear clinical guidelines are lacking. Moreover, this recommendation has been challenged by the conflicting results of randomized controlled trials published so far [4, 7–9].

Among the available options, funnel-shaped meshes have gained attention due to encouraging results from observational studies [10–14] and one randomized controlled trial [15]. These meshes are specifically designed to facilitate proper positioning of the bowel and to minimize the risk of creating an orifice that is either too large—predisposing to herniation—or too small, which may increase the risk of bowel strangulation. Additionally, funnel meshes are intended to stabilize the intestinal loop by securing it to the abdominal wall, thereby limiting its mobility through the stoma site. However, robust evidence from well-designed trials is still required to validate their role in PSH prevention.

The aim of this study is to investigate the incidence of PSH and postoperative complications after APR with permanent terminal colostomy using the keyhole technique with prophylactic placement of a funnel-shaped 3D mesh (DynaMesh^®^-IPST).

## Materials and methods

This study is reported in accordance to the Strengthening the Reporting of Observational Studies in Epidemiology (STROBE) guidelines [16].

### Study design and setting

A retrospective cohort study was conducted using data from a prospectively maintained institutional database of the Department of Minimally Invasive Surgical Oncology, ASST Grande Ospedale Metropolitano Niguarda, Milan, Italy. The study period was from April 2011 to May 2019. In May 2015, the department introduced the systematic use of intraperitoneal prophylactic mesh placement during elective PEC construction.

According to national regulations and institutional policy, retrospective studies based on anonymized data from prospectively maintained clinical databases do not require specific approval by the Ethics Committee. All procedures were conducted in accordance with the Declaration of Helsinki, and all patients provided general informed consent for the use of their clinical data for research purposes.

### Participants

All adult patients who underwent elective PEC for any indication between April 2011 and May 2019 were eligible for participation. The control group (without mesh) comprised patients who underwent surgery between April 2011 and April 2015, i.e. before the introduction of the prophylactic mesh. The mesh group included patients treated between May 2015 and May 2019, when prophylactic meshes were systematically introduced. Exclusion criteria included emergency surgery, open approach, temporary stoma formation or incomplete follow-up data.

### Intervention

In the mesh group, a three-dimensional, funnel-shaped, two-component mesh (DynaMesh^®^-IPST) consisting of polyvinylidene fluoride (PVDF, 85%) and polypropylene (PP) was used. The parietal surface (PVDF with PP) was characterised by green threads, while the visceral surface consisted entirely of PVDF. The meshes were placed intraperitoneally using a standardised laparoscopic technique and fixed with absorbable tacks and N-butyl-2-cyanoacrylate glue.

### Outcomes and definitions

The primary outcome was the incidence of PSH, defined according to the European Hernia Society (EHS) classification [4]. PSH diagnosis was based on both clinical examination, performed at 1, 3, and 12 months postoperatively, and radiological assessment. All patients underwent a computed tomography (CT) scan at 12 months as part of routine oncological follow-up. In cases where further imaging was indicated (e.g., ultrasound or magnetic resonance imaging), results were included to confirm or rule out PSH. Only radiologically confirmed PSH were considered for the primary analysis to reduce potential diagnostic bias from clinical examination alone.

### Follow-up examination

Patients underwent physical examinations at 1, 3 and 12 months postoperatively, including assessment of the Valsalva manoeuvre. Imaging examinations were performed in case of clinical suspicion or as part of routine oncological surveillance, with a mandatory CT scan at 12 months.

### Statistical analysis

No formal sample size calculation was performed given the retrospective, observational nature of the study; however, all eligible consecutive patients during the study period were included to maximize statistical power.

Quantitative data are reported as medians with ranges, while categorical variables are presented as absolute numbers and percentages. Between-group comparisons for categorical variables were performed using Fisher’s exact test or Chi-Square test, as appropriate. Continuous variables were compared using the Mann-Whitney U test. A two-sided p-value < 0.05 was considered statistically significant.

The probability of PSH occurrence over time (expressed in months) was estimated using Kaplan-Meier survival analysis, and differences between groups were assessed with the log-rank test. Multivariable Cox proportional hazards regression was performed to adjust for potential confounding factors associated with PSH development.

In addition, a post hoc power analysis was planned for the primary binary endpoint (radiologically confirmed PSH). The analysis was conducted using the observed proportions of PSH in the two groups, with a two-sided significance level of 0.05. Effect size was expressed as Cohen’s h, and the achieved statistical power as well as precision-based estimates (absolute risk difference and risk ratio with 95% confidence intervals) were reported. For interpretative purposes, the required sample size to achieve conventional power levels (80% and 90%) was also estimated based on the observed effect size.

## Results

Between April 2011 and May 2019, 88 patients underwent elective APR for low rectal cancer with PEC construction at our institution. Of these, a total of 13 patients were excluded from the analysis. Specifically, six patients required conversion to open surgery due to anesthesiological contraindications to a minimally invasive approach. Three patients were excluded because of intraoperative contraindications to mesh placement, such as accidental bowel perforation with a subsequent risk of mesh contamination. Finally, four patients were excluded due to missing data.

**Fig. 1:**
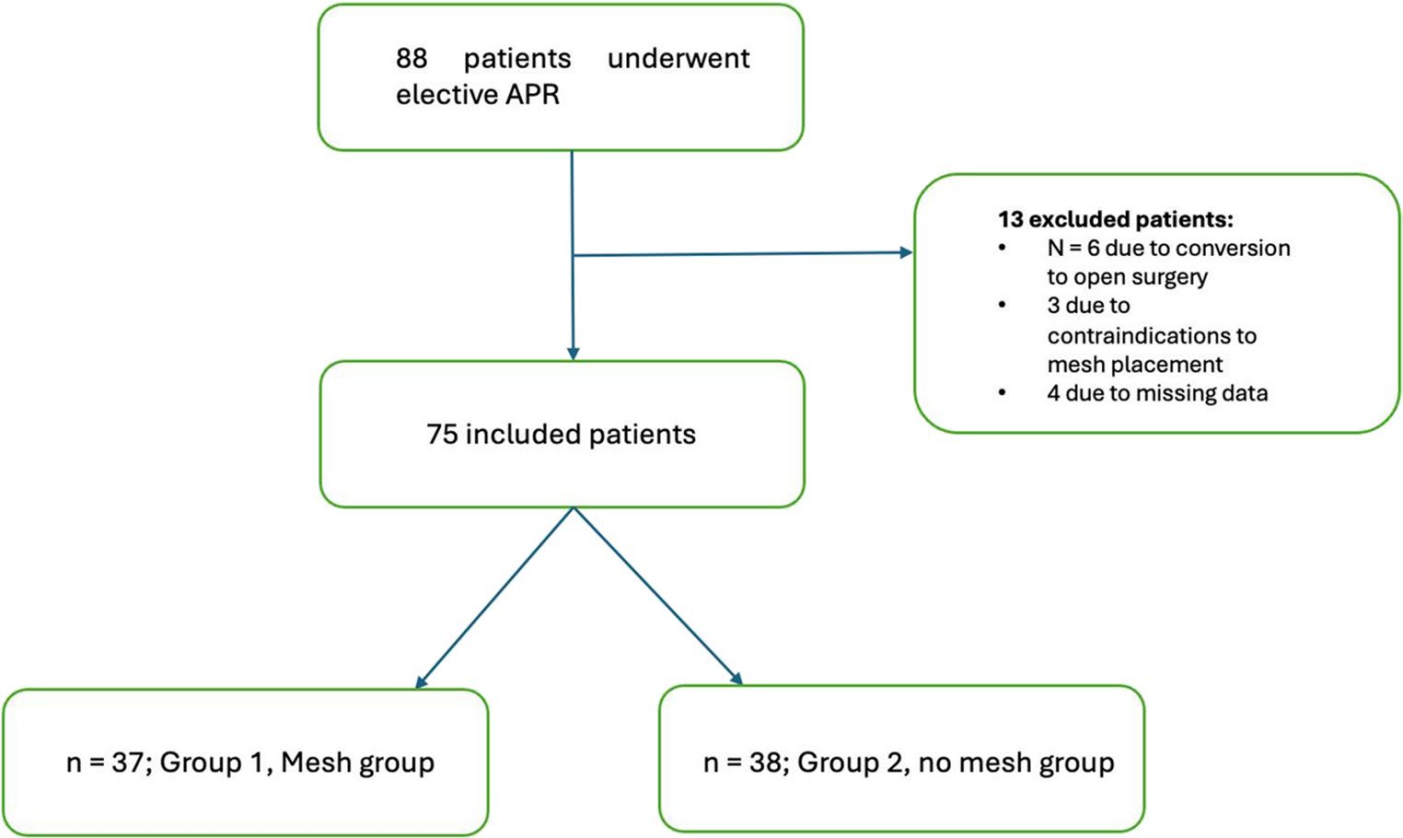
Flow diagram illustrating the number of patients assessed for eligibility, excluded (with reasons), and included in the final analysis, following STROBE-recommended reporting

Therefore, a total of 75 patients were included in the study. Of these, 37 patients were included in the mesh group (Group 1) and received prophylactic placement of a three-dimensional funnel-shaped mesh (DynaMesh^®^-IPST), while 38 patients formed the no-mesh group (Group 2) and underwent colostomy construction without mesh placement (Fig. 1).

### Baseline characteristics

Table 1 summarizes the demographic and clinical characteristics of the two groups. No statistically significant differences were found between groups in terms of age, sex, body mass index (BMI), comorbidities, smoking status, prior abdominal surgery, or neoadjuvant/adjuvant treatments. Additionally, no patients in either group were classified as ASA IV.

**Table 1:** Demographic characteristics of the study population

	Total, *n* (%) 66	Group 1 (Mesh), 37 *n* (%)	Group 2 (No mesh) 38 *n* (%)	*p* value
Age (median)		67	69	
Female		16 (43.24)	13 (34.2)	
Male		20 (54.05)	25 (65,8)	
BMI (median)		25	24 (63,2)	*0.2344*
OBESITY	9	6 (16,22)	3 (7.9)	*0.1229*
DM	14	9 (24,3)	5 (13,2)	*0.1539*
ASA				*0.1118*
I	9	4 (10,81)	5 (13,2)	
II	49	25 (67,57)	24 (63,2)	
III	16	8 (21,6)	8 (21,1)	
Glucocorticoids	0	0	0	
Immunosuppressant	2	1 (2.7)	1 (2,6)	*0.9999*
Previous abdominal surgery	30	16 (43,3)	14 (36,8)	*0.3201*
Smoke	31	17 (45,9)	14 (36,8)	*0.3719*
Neoadjuvant therapy	42	24 (64,8)	18 (47,4)	*0.196*

The median follow-up time was 46 months (IQR: 48 months) in the mesh group and 43 months (IQR: 44 months) in the no-mesh group. All procedures were performed laparoscopically using a standardized surgical technique previously described [10].

### Postoperative complications and adjuvant therapy

Postoperative complications, classified according to Clavien-Dindo [17], and the administration of adjuvant therapy are reported in Table 2. No statistically significant differences were found between the two groups in the complication rates (*p* = 0.2305) and in the administration of adjuvant therapy (*p* = 0.1422).

**Table 2:** Postoperative complications and patients who received adjuvant therapy

	Total, *n* (%)	Group 1 (Mesh), *n* (%)	Group 2 (No mesh), *n* (%)	*p* value
Adjuvant therapy	45	23 (62,1)	22 (57,9)	*0.1422*
Clavien Dindo				*0.2305*
0	40	19 (51,3)	21 (55.3)	
1	28	13 (35,4)	15 (39,5%)	
2	1	1 (2,7)	0	
3	3	3 (8,1)	0	
4	2	1 (2,7)	1 (2,6)	
5	1	0	1 (2,6)	

However, in the mesh group:

- One patient developed a mesh-related small bowel obstruction that required early reoperation with laparotomy, bowel resection and mesh removal.
- Another patient developed a mesh infection two years postoperatively, necessitating mesh explantation.

### Parastomal hernia incidence

The primary outcome, radiologically confirmed PSH, was assessed using CT in 98% of patients and ultrasound in 2% in combination with clinical evaluation. PSH incidence was lower in the mesh group compared to the no-mesh group, although not statistically significant (21.6% vs. 39.5%; *p* = 0.094).

**Fig. 2:**
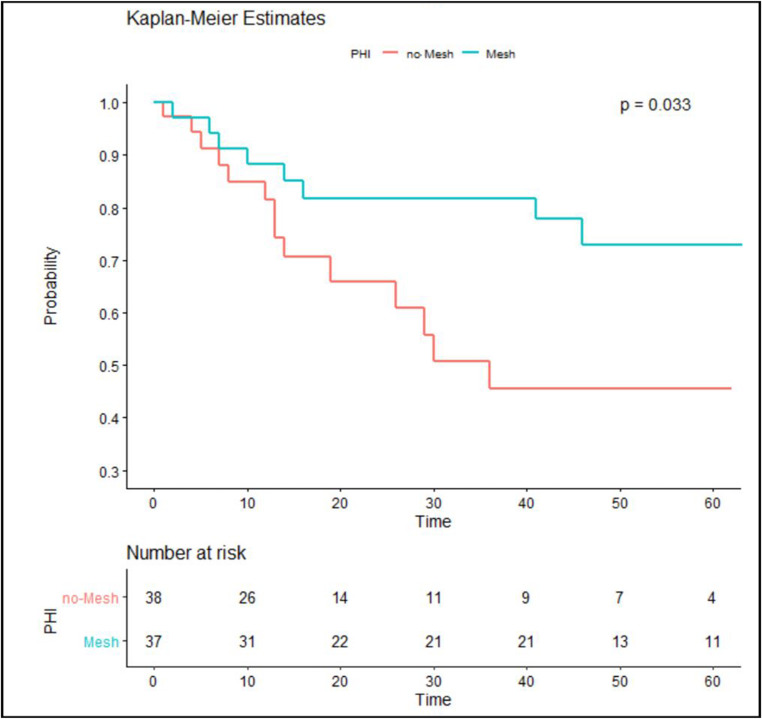
Kaplan–Meier curve showing the time to occurrence of radiological parastomal hernia (PSH)

The Kaplan-Meier survival analysis (Fig. 2) showed that:

- In the mesh group, over 80% of patients remained hernia-free at 40 months of follow-up.
- In the no-mesh group, only 55% of patients remained hernia-free at the same timepoint.

The cumulative incidence over time in PSH incidence between groups at 40 months was statistically significant (*p* = 0.033). The protective effect of the prophylactic funnel-shaped mesh became clearer after the first 10 months postoperatively and was also maintained in the long-term course.

### Post hoc power analysis

A post hoc power analysis was performed for the primary binary endpoint (radiologically confirmed PSH). Based on the observed proportions (21.6% in the mesh group [8/37] vs. 39.5% in the no-mesh group [15/38]), the achieved statistical power was approximately 39–40% (Cohen’s h = 0.391, two-sided α = 0.05). The absolute risk difference was − 17.9% (95% CI − 43.9% to + 11.6%), and the risk ratio was 0.55 (95% CI 0.26–1.14). Under the same effect size, an equal-allocation study would require approximately 205 patients to reach 80% power and 274 patients for 90% power.

## Discussion

This retrospective cohort study shows that prophylactic placement of a three-dimensional funnel-shaped mesh during PEC creation is associated with a significant reduction in the cumulative risk of PSH over time, as shown by Kaplan-Meier analysis. Although the absolute difference in PSH incidence between the groups did not reach conventional statistical significance, time-to-event analysis showed a clinically meaningful benefit, with the divergence of survival curves after the first postoperative year becoming apparent. This result emphasizes the importance of longer follow-up in the evaluation of interventions for PSH, a complication that often manifests itself in the medium to long term.

The role of prophylactic mesh in the prevention of PSH is the subject of ongoing debate. Although both the 2018 European Hernia Society (EHS) guidelines [4] and a Cochrane review of randomized controlled trials (RCTs) [18] advocate the placement of mesh during elective stoma creation, its use in practice remains limited and the implementation rate is below 10% [10].

The STOMAMESH trial—the largest multicenter RCT conducted to date—did not demonstrate a significant reduction in PSH rates with prophylactic mesh [7]. Importantly, this trial combined elective and emergency cases in the analysis, introducing heterogeneity that may have obscured potential subgroup-specific benefits. Moreover, the mesh design employed—a flat prosthesis with a central aperture—primarily reinforced the abdominal wall but did not address stoma loop mobility, a key factor implicated in PSH pathophysiology.

Recent observational studies, a randomized controlled trial and meta-analyses suggest that funnel-shaped meshes, specifically designed to stabilise the bowel loop and minimise its mobility, may provide better protection against PSH [11, 15]. For example, Conde-Muíño et al. [14] reported a PSH rate of 6.5% when using funnel meshes, while a recent meta-analysis found a significant reduction in PSH incidence (9% versus 54%) compared to controls. However, most of the available data are from observational studies with limited follow-up, and the only RCT included in the meta-analysis had a relatively short duration. In fact, In the Chimney trial, Mäkäräinen et al. reported a parastomal hernia (PSH) rate of 10% in the funnel mesh group (6/58) compared to 37% in the no-mesh group (22/59), based on a 12-month radiological follow-up using CT scan [15].

Berger et al. [19] documented no cases of PSH following prophylactic implantation of a funnel-shaped mesh, with a median follow-up period of 11 months. A recent systematic review and meta-analysis reinforced these findings, demonstrating significantly lower rates of PSH in patients receiving prophylactic funnel-shaped mesh compared to controls (9% vs. 54%; OR 0.07; 95% CI 0.03–0.18; *p* < 0.001) [20]. Notably, these results were observed over a mean follow-up period of 37.0 ± 16.4 months, providing mid-term evidence for the effectiveness of this prophylactic approach. However, these results should be considered exploratory, as the majority of the included studies were observational in nature, and the only randomized controlled trial included had a follow-up period of just 12 months.

Traditional flat meshes with a central aperture are intended to reinforce the abdominal wall but fail to stabilize the stoma loop, a critical factor in the development of parastomal hernia (PSH). Longer-term data suggest that while these meshes may delay hernia onset, they do not consistently prevent its occurrence [21]. To overcome these limitations, three-dimensional funnel-shaped meshes were introduced, designed to better distribute tension around the stoma and limit bowel mobility. Early observational studies have reported encouraging reductions in PSH rates with this approach; however, concerns persist that these devices may simply postpone, rather than definitively prevent, hernia formation. Our findings align with this hypothesis. Although the absolute difference in PSH incidence between groups did not reach conventional statistical significance, Kaplan-Meier analysis demonstrated a significant and sustained reduction in cumulative PSH risk over time among patients receiving prophylactic funnel mesh. Notably, the survival curves diverged beyond the first 10 postoperative months, suggesting that the mesh effect becomes more evident in the medium-to-long term. Considering that PSH often presents as a delayed complication, this temporal impact is clinically relevant, as postponing hernia onset may improve quality of life and reduce the need for early reinterventions. Importantly, our study addresses a recognized limitation of previous research by providing extended follow-up, with a median of 46 months in the mesh group and 43 months in controls, offering robust mid- to long-term evidence. Furthermore, we observed no significant increase in postoperative complications, supporting the safety profile of prophylactic mesh placement when appropriately selected.

### Strengths

Key strengths of this study include the use of a standardized surgical protocol and the exclusive inclusion of minimally invasive procedures, which together ensure a high degree of procedural consistency and reproducibility. The use of a single, well-characterized funnel-shaped mesh also minimized heterogeneity and allowed for a targeted evaluation of this specific procedure. The extended follow-up period − 46 months on average in the mesh group — enabled robust detection of late-onset parastomal hernias, overcoming a major limitation of previous studies with shorter observation periods and providing valuable medium- to long-term outcome data.

In addition, the use of radiologically confirmed PSH as the primary endpoint minimized the risk of diagnostic bias associated with clinical examination alone, thereby increasing the reliability of outcome assessment. The inclusion of consecutive patients from a prospectively maintained database reduced the risk of selection bias and improved the completeness and accuracy of the clinical data. In addition, the study’s comprehensive follow-up protocol, which included both scheduled imaging and clinical assessments, ensured systematic and thorough monitoring of all participants.

Taken together, these methodological strengths contribute to the robustness and clinical relevance of the study’s conclusions.

### Limitations

Several limitations of this study should be considered. First, the retrospective design is inherently susceptible to selection bias and confounding, which limits the ability to establish definitive causal relationships between prophylactic mesh insertion and the occurrence of PSH. Second, loss to follow-up, primarily due to mortality from advanced malignancies, may have led to an underestimation of late PSH incidence, particularly in a population with limited long-term survival. In the no mesh group, shorter follow-up was mainly explained by censoring events, as 6 patients died from cancer-related causes and 14 developed a parastomal hernia, resulting in early termination of observation for more than half of the group.

Third, the fact that the study was conducted at a single center may limit the external validity of our results, as institutional protocols, patient demographics and surgical expertise may differ from those at other institutions. Fourth, the lack of randomization increases the risk of unmeasured confounding variables influencing the observed results. Finally, the relatively modest sample size, although including all eligible cases over an extended period of time, limits the statistical power to detect small but clinically important differences and may preclude robust subgroup analyses.

Moreover, potentially relevant parameters such as waist circumference, stoma aperture size, and standardized measures of nutritional status were not systematically collected in our retrospective dataset. These unmeasured confounders may have influenced the results and should be incorporated in future prospective analyses.

Additionally, the limited sample size of our cohort resulted in a low post hoc statistical power for the fixed-time binary comparison of PSH incidence. This explains why the absolute incidence difference did not reach statistical significance despite favoring the mesh group. Importantly, the time-to-event analysis, which accounts for censoring due to mortality or early PSH diagnosis, demonstrated a significant cumulative benefit of prophylactic funnel mesh. Taken together, these findings suggest a time-dependent protective effect, while underscoring the need for larger, adequately powered prospective trials to confirm our results.

Future multicenter, prospective, randomized controlled trials with larger sample sizes and longer follow-up are warranted to validate these findings, improve generalizability and better inform clinical practice.

## Conclusions

Prophylactic placement of a three-dimensional funnel-shaped mesh during PEC construction appears to reduce the cumulative incidence of PSH without increasing postoperative morbidity. These results provide additional medium- to long-term evidence in favor of the use of funnel-shaped meshes in this area. Nevertheless, large-scale, multicenter, randomized controlled trials are needed to confirm these results, refine patient selection criteria and support the development of evidence-based clinical guidelines for the prevention of PSH.
